# Silencing an essential gene involved in infestation and digestion in grain aphid through plant‐mediated RNA interference generates aphid‐resistant wheat plants

**DOI:** 10.1111/pbi.13067

**Published:** 2019-02-06

**Authors:** Yongwei Sun, Caroline Sparks, Huw Jones, Mandy Riley, Frédéric Francis, Wenming Du, Lanqin Xia

**Affiliations:** ^1^ Institute of Crop Sciences Chinese Academy of Agricultural Sciences (CAAS) Beijing China; ^2^ Functional and Evolutionary Entomology Gembloux Agro‐bio Tech University of Liege (ULg) Gembloux Belgium; ^3^ Rothamsted Research Harpenden, Herts UK; ^4^ Institute of Biological, Environmental & Rural Sciences (IBERS) Aberystwyth University Aberystwyth UK

**Keywords:** grain aphid (*Sitobion avenae* F.), RNA interference (RNAi), wheat (*Triticum aestivum* L)

## Conflict of interest

The authors have filed a patent application based on the studies in this paper.

## Authors’ contributions

L.Q.X. conceived the project. L.Q.X., H. J. and F. F. designed the experiments. Y.W.S., C.S., M.R. and W.M.D did the experiments. L.Q.X. and Y.W.S. wrote the manuscript. All authors read and approved the manuscript.

Dear Editor,

Aphids are major agricultural pests which cause significant yield losses of crop plants each year by inflicting damage, both through the direct effects of feeding and by vectoring debilitating plant viruses. In the absence of genetic plant resistance, insecticide treatments remain the main means for aphid control. However, excessive dependence on insecticides is undesirable because of the development of insecticide resistance, the potential negative effects on non‐target organisms and environmental pollution (Yu *et al*., 2014). Transgenic plants expressing dsRNAs designed against insect target genes are emerging as a next generation of pest management strategy with benefits over chemical‐based pesticides or traditional biological control because the sequence‐specific nature of RNAi allows for individual species and potentially specific orders of pests to be selectively targeted (Abdellatef *et al*., 2015; Coleman *et al*., 2015; Mao *et al*., 2007; Price and Gatehouse, 2008; Whyard *et al*., 2009). For the successful application of plant‐mediated RNAi for aphid control in wheat, identification of effective species‐specific RNAi targets is the prerequisite. However, not all the target genes are sensitive to dsRNA treatment. Of the major aphid species infesting wheat (*Triticum aestivum* L.), grain aphid (*Sitobion avenae* F.) is the most dominant and destructive and major pest of wheat in China, Europe and North America (Yu *et al*., 2014). In our previous study, among a set of 66 unigenes involved in different biological processes in grain aphid and selected for a dsRNA artificial diet assay, only four effective RNAi targets were exploited (Wang *et al*., 2015). We also performed *de novo* transcriptome assembly and gene expression analyses of the alimentary canals of grain aphids before and after feeding on wheat plants using Illumina RNA sequencing. Based on the RPKM values of these unigenes, 16 of them that were significantly up‐ or down‐regulated upon feeding were selected for dsRNA artificial diet feeding assay. Among these genes, only five potential RNAi targets were identified (Zhang *et al*., 2013).

**Figure 1:**
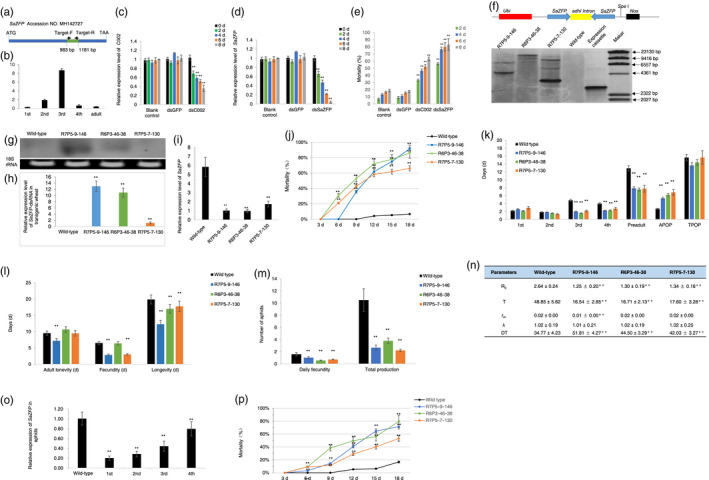
Silencing a *SaZFP* gene by plant‐mediated RNAi for wheat protection against aphid infestation. (a) A schematic description of the cDNA *of SaZFP*, the locations of the fragment for making RNAi construct and primers used to evaluate the expression of *SaZFP*. The NCBI Genbank accession number is MH142727. The fragment used for making RNAi construct from 983 to 1181 bp is highlighted in green, and the locations of primer set Target‐F and Target‐R are from 983–1009 bp and 1157–1181 bp, respectively. (b) The expression profile of *SaZFP* in grain aphid at different development stages. (c) The expression level of *C002* in the third instar aphids fed on an artificial diet supplemented with *C002‐*dsRNA. (d) The expression level of *SaZFP* in the third instar aphids fed on an artificial diet supplemented with *SaZFP‐*dsRNA. (e) The mortality of third instar grain aphids fed on an artificial diet supplemented with *SaZFP‐*dsRNA at different time points. (f) A schematic show of the *SaZFP*‐dsRNA expression cassette and position of *Spe* I restriction enzyme. Genomic DNA was digested with *Spe* I and hybridized with a *SaZFP* gene fragment with the expression cassette digested with *Spe* I as a positive control. (g) Northern blot of the *SaZFP*‐dsRNA in transgenic wheat lines. (h) The relative expression levels 2nd of *SaZFP*‐dsRNA in different transgenic wheat lines. (i) Relative expression levels of *SaZFP* of grain aphids fed on wild‐type and transgenic wheat lines. (j) Mortality of aphids fed on wild‐type and transgenic wheat lines. (k) The longevity of different stages, adult preoviposition period (APOP) and total preoviposition period (TPOP) of aphids fed on transgenic lines and wild‐type control. (l) The adult longevity, fecundity and the total longevity of aphids fed on transgenic wheat lines and wild‐type control. (m) The reproduction of aphids fed on transgenic wheat lines and the wild‐type control. (n) Life table parameters of aphids fed on wild‐type and different transgenic wheat lines. *R*_0_, net reproductive rate; *r*_m_, the intrinsic rate of increase; λ, the finite rate of increase; *T*, the mean generation time; DT, Doubling time (day). (o) The *SaZFP* transcript levels of third instar aphids in four successive aphid generations. (p) The mortality of the first generation of the offspring of aphids fed on transgenic lines at different time points after being switched to wild‐type plants. Values and bars represent the mean ± SEM of three independent biological replicates (Student's *t*‐test, ** *P *< 0.01).

Based on our previous study, here we isolated and characterized a novel gene from grain aphid which is significantly up‐regulated upon feeding on wheat plants (Genbank accession number is MH142727). This gene is 1368 bp in length, encoding a 455 amino acids protein which is predicted to encode an ortholog of a zinc finger protein in pea aphid (http://bipaa.genouest.org/is/aphidbase/). We designated this gene as *SaZFP* (a zinc finger ortholog in *S. avenae* F.). Then, the expression profiles of *SaZFP* at different developmental stages were investigated by quantitative real‐time PCR (qRT‐PCR) with primer set Target‐F/Target‐R (Figure 1a). We found that *SaZFP* transcripts accumulated at different levels throughout the developmental stages. The expression pattern of *SaZFP* was up‐regulated, and peaked in the third instar nymphs, then decreased gradually from the fourth instar stage (Figure 1b).

To evaluate the functional role of *SaZFP* in aphid development and survival, we first performed *in vitro* dsRNA synthesis by using a 198 bp fragment of *SaZFP* gene as a template. To check the specificity of this gene fragment to be utilized as an RNAi target in grain aphid, we used Basic Local Alignment Search Tool (BLAST) against NCBI database (www.ncbi.nlm.nih.gov/blast). The result revealed that no continuous three 21‐nt matches exist between this 198 bp fragment and aphid predators or parasitoids at the nucleotide acid level, indicating that the dsRNAs derived from the coding sequences of *SaZFP* would be potentially safe for non‐target organisms (Bachman *et al*., 2013). Then, we did the *in vitro* dsRNA artificial diet feeding assay by using the dsRNA of green fluorescent protein (GFP) gene as a negative control and the dsRNA of *C002* in grain aphid, which was homologous to *C002*, a salivary protein encoding gene in pea aphid (Mutti *et al*., 2008), as a positive control in the dsRNA feeding experiments (Figure 1c). We observed that ingestion of *SaZFP*‐dsRNA resulted in a significant decrease in *SaZFP* mRNA level from the 2nd day after feeding (DAF; Figure 1d). At the 8th DAF, the *SaZFP* transcripts abundance in the aphids fed with *SaZFP*‐dsRNA were significantly lower compared to that of aphids fed on diet without dsRNA (blank control) (*P *< 0.01), whereas no obvious variations were detected between the transcript abundance of *SaZFP* in *GFP*‐dsRNA feeding aphids and that of the blank control (Figure 1d). Knock‐down of *SaZFP* expression by dsRNA feeding led to significant mortality and developmental stunting of aphids. As indicated in Figure 1e, *SaZFP*‐dsRNA feeding resulted in a mortality around 50% at the 2nd day, reaching 80% at the 8th day, which was even higher than treatment with *C002*‐dsRNA (Mutti *et al*., 2008), and significantly higher than the control treatments with *GFP*‐dsRNA or blank control (*P *< 0.01; Figure 1e). Compared with the blank control, the *GFP*‐dsRNA in the artificial diet had no effect on the mortality of aphids. Thus, the lethality observed in the *SaZFP*‐dsRNA feeding group indicated a sequence‐specific effect of the dsRNA rather than physical/chemical characteristics of dsRNAs per se.

We then transformed wheat immature embryos (*Triticum aestivum* L. *cv* Cadenza) with the vector harbouring Ubi‐*SaZFP*‐hairpin DNA in which the maize ubiquitin promoter (*Ubi*) was used to drive the constitutive expression of sense and antisense copies of a 198 bp fragment of *SaZFP* to generate the corresponding dsRNA. In total, 22 transgenic lines were obtained. Among them, three transgenic lines R7P5‐9‐146, R6P3‐46‐38 and R7P5‐7‐130 were randomly selected for further analyses. Southern blot showed that the *SaZFP*‐dsRNA expression cassette had been stably integrated into the wheat genome with two to four copies respectively (Figure 1f). The expression levels of *SaZFP*‐dsRNA in different lines were then evaluated by northern blot analysis (Figure 1g) and qRT‐PCR (Figure 1h), respectively. The results showed that line R7P5‐9‐146 had a higher dsRNA expression level than other two lines (Figure 1g and h).

We further investigated whether the expression of the inhibitory dsRNA affected target gene expression in aphids fed on transgenic wheat lines. The synchronous 1‐day‐old nymphs were transferred to transgenic wheat lines and wild‐type wheat plants respectively. After several days, the third instars were selected to investigate the relative expression levels of *SaZFP*. As indicated in Figure 1i, the relative expression levels of *SaZFP* in aphids fed on three transgenic lines decreased at a significant level (*P *< 0.01) compared to those aphids on wild‐type plants.

We then evaluated the impact of *SaZFP* silencing on various aphid fitness parameters such as mortality, life cycle and population development parameters by challenging the different transgenic lines with grain aphids. For each experiment, the synchronous 1‐day‐old nymphs that were born on aphid‐susceptible wheat plants (*cv* Beijing 837) were isolated individually into clip cages on transgenic and wild‐type wheat plants. The mortality of aphids fed on the transgenic lines increased significantly compared with wild‐type control at 6th DAF, and at 18th DAF, the mortalities reached more than 80% (Figure 1j). The aphid development duration from the newborn nymphs until imago stage was monitored. Compared with those of aphids fed on the wild type, the longevity of different stages of aphids fed on transgenic wheat lines was significantly shortened, whereas the adult preoviposition period (APOP) extended in transgenic lines significantly (*P *< 0.01) (Figure 1k). We also found the total longevity and period of duration of aphids fed on transgenic wheat lines significantly decreased compared to these on the wild type (*P *< 0.01) (Figure 1l). As a result, the daily fecundity and total production of aphids fed on transgenic lines decreased at a significant level (*P *< 0.01) (Figure 1m). Further evaluation of the population parameters showed that the net reproductive rate (*R*_0_) and doubling times of the population (DT) were significantly lower than those on wild‐type plants, whereas the mean generation time (*T*) extended (*P *< 0.01) (Figure 1n).

We also investigated the potential transgenerational RNAi effects of *SaZFP* in a parallel experiment. The 1‐day‐old newborn nymphs on transgenic wheat lines were transferred to fresh wild‐type wheat plants and subsequently allowed to reproduce on wild‐type plants, and then the expression level of *SaZFP* was investigated. Remarkably, the expression of *SaZFP* in grain aphids was not only inhibited in the parental lines that fed on transgenic lines, but also remained significantly reduced in the subsequent generations (Figure 1o). Aphids recovered slowly from *SaZFP* silencing over successive generations, with relative expression levels reaching 20.3%, 28.6%, 44.3% and 79.8% of control levels in successive 1st–4th generations respectively (Figure 1o). The offspring of aphids fed on transgenic lines still displayed higher mortality rates even after being switched to wild‐type plants (Figure 1p).

Taken together, we here not only identified a novel potential RNAi target gene *SaZFP* which is involved in ingestion and digestion from grain aphid, but also illustrated that targeted silencing of *SaZFP* in grain aphids through plant‐mediated RNAi significantly reduced the fecundity, survival and total production of aphids, thus minimizing aphid infestation on wheat plants. The observed parental and transgenerational RNAi effects should also lead to decreased aphid infestation on wheat plants. Therefore, our results demonstrate the substantial potential of plant‐mediated RNAi of an essential gene involved in ingestion and digestion as an alternative strategy for aphid management in agriculture practice.
